# Comparison between single-marker analysis using Merlin and multi-marker analysis using LASSO for Framingham simulated data

**DOI:** 10.1186/1753-6561-3-s7-s27

**Published:** 2009-12-15

**Authors:** Yun Ju Sung, Treva K Rice, Gang Shi, C Charles Gu, DC Rao

**Affiliations:** 1 Division of Biostatistics, Washington University School of Medicine, 660 South Euclid Avenue, Box 8067, St. Louis, Missouri 63110-1093, USA; 2 Department of Psychiatry, Washington University School of Medicine, 660 South Euclid Avenue, Box 8067, St. Louis, Missouri 63110-1093, USA; 3 Department of Genetics, Washington University School of Medicine, 660 South Euclid Avenue, Box 8067, St. Louis, Missouri 63110-1093, USA

## Abstract

We compared family-based single-marker association analysis using Merlin and multi-marker analysis using LASSO (least absolute shrinkage and selection operator) for the low-density lipoprotein phenotype at the first visit for all 200 replicates of the Genetic Analysis Workshop 16 Framingham simulated data sets. Using "answers," we selected single-nucleotide polymorphisms (SNPs) on chromosome 22 for comparison of results between single-marker and multi-marker analyses. For the major causal SNP rs2294207 on chromosome 22, both single-marker and multi-marker analyses provided similar results, indicating the importance of this SNP. For the 12 polygenic SNPs on the same chromosome, both single-marker and multi-marker analyses failed to provide statistically significant associations, indicating that their effects were too weak to be detected by either method. The main difference between the two methods was that for the 14 SNPs near the causal SNPs, *p*-values from Merlin were the next smallest, whereas LASSO often excluded these non-causal neighboring SNPs entirely from the first 10,000 models.

## Background

Association analysis is often performed using single markers or haplotype analysis of multiple single-nucleotide polymorphisms (SNPs) within adjoining short regions or candidate genes. However, analysis that simultaneously uses multiple markers may be more powerful for detecting several causal genes and, hence, may be more appropriate for complex diseases [1].

The least absolute shrinkage and selection operator (LASSO) is a penalized least squares method imposing the L1-penalty on the regression coefficients [2]. Because this penalty induces shrinkage, prediction using LASSO is more reproducible than the regular multiple linear regression, in the case when there are more predictors than individuals (small *n *large *p*). Compared with a regular multiple linear regression (ordinary least squares), LASSO can handle the multicollinearity resulting from the highly correlated markers. Moreover, due to the nature of the L1-penalty, many regression coefficients are exactly zero. Hence, LASSO does both shrinkage and automatic variable selection simultaneously, a form of parsimonious model selection.

Our main goal in this paper was to explore the performance of LASSO for SNP selection in association analysis. In particular, we compared the relative importance (ranks) of SNPs provided by LASSO to that of SNPs inferred by single-marker analysis.

## Methods

### Phenotypes and genotypes

We used the low-density lipoprotein (LDL) phenotype at the first visit for all 200 replicates of the Genetic Analysis Workshop 16 (GAW16) Framingham simulated data sets. This phenotype was adjusted for age, smoking, and diet separately for both sexes and then corrected for medication (HMG-CoA reductase inhibitors) [3]. Because the GAW16 data set only contained individuals with genotypes, we created records for untyped parents as founder individuals. Because their actual relationship with other members in the same family ID was not provided, one extended family was often divided into multiple families: 1129 families with size ranging from 1 to 470 became 1920 families with size ranging from 1 to 72. Chromosome 22 included one major causal SNP and 12 polygenic SNPs that influenced the simulated LDL phenotype [4]. To reduce the number of SNPs, we chose 5011 SNPs located between 23.28 Mb and 49.10 Mb, 0.1 Mb in each direction past the left and right influencing SNPs. We excluded SNPs with minor allele frequency (MAF) less than or equal to 0.003 (we wanted to include one polygenic SNP with MAF 0.004). The final data set for analysis consisted of 4589 SNPs and 6857 individuals.

### Single-marker analysis using Merlin

For single marker analysis, we used Merlin [5,6]. The family-based association test provided by Merlin has two advantages. First, missing genotypes (1.5% of all genotypes) were imputed, using flanking markers and family relationships, and incorporated in the association test. Second, unlike most family-based linkage and association programs, which do not provide results for data sets with mendelian inconsistent genotypes, the Merlin association test does provide results by ignoring families with mendelian inconsistent genotypes. Even though this may not be an optimal way to handle genotype errors, it bypasses removing genotype errors, which can be tedious for data sets with large number of SNPs and large families. Linkage disequilibrium (LD) between the major causal SNP and other SNPs (measured by *r*^2^) was computed using R package genetics.

### Multi-marker analysis using LASSO

For covariate-adjusted phenotype *y*_*i *_and SNPs *x*_*i*1_,..., *x*_*ip *_of *i*^th ^individual, LASSO minimizes  subject to . The LASSO solution path provides a sequence of models, from the simplest model including only an intercept (when *t *= 0) to the most complex model including all SNPs as predictors (when *t *is very large). If a particular SNP becomes a predictor in the *i*^th ^model, then that SNP tends to stay as a predictor for all bigger models, but this does not always happen. For ranking SNPs, we used this "entry" number that indicates when a particular SNP becomes a predictor in the LASSO solution path. For our analysis, we evaluated the first 10,000 models in the LASSO solution path, using R package lars [7]. We used Merlin to impute missing SNPs because lars requires each individual to have values for all predictors: removing individuals with partially missing SNPs would make use of only one-tenth of the data. This also makes the data set more consistent with single-marker analysis.

## Results

### Single-marker analysis using Merlin

**Figure 1:**
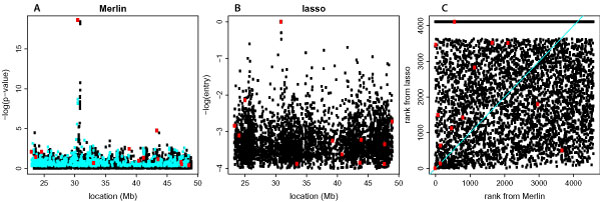
**Association tests of 4589 SNPs on chromosome 22 for Replicate 1 of the simulated LDL phenotype**. A, *p*-values from single-marker analysis using Merlin; B, entry numbers from multi-marker analysis using LASSO; C, comparison of ranks from Merlin and LASSO (correlation = 0.08). Red dots indicate 1 major causal SNP and 12 polygenic SNPs. Cyan points in A indicate 960 SNPs that were not in any of the first 10,000 models from LASSO.

Figure 1A shows association test results for Replicate 1 of 200 simulated LDL phenotypes: results were consistent across all 200 Replicates (Table 1). The major causal SNP rs2294207 provided statistically significant association with *p*-value 4.5 × 10^-19 ^for Replicate 1: for all 200 replicates, this SNP ranked 1.1 on average (Table 1) with *p*-values ranging from 6.9 × 10^-13 ^to 1.6 × 10^-29^. In Replicate 1, 14 SNPs near the major causal SNP (10 SNPs around 30.91 and 4 SNPs around 30.95) had *p*-values ranging from 3.0 × 10^-8 ^to 3.8 × 10^-19 ^(Figure 1A): these SNPs provided significant association across all 200 replicates (Table 1). Ranks of these neighboring SNPs were almost in the order of LD between them and the causal SNP. Out of 12 polygenic SNPs, the most significantly associated SNP was rs5765113 (*p*-value 3.5 × 10^-5 ^ranking 20 for Replicate 1): for all 200 replicates, this SNP ranked 35.8 on average (Table 1) with *p*-values ranging from 5.7 × 10^-2 ^to 7.9 × 10^-8^.

**Table 1:** Summary statistics, based on Replicates 1 through 200, for 12 polygenic SNPs and SNPs near the major causal SNP rs2294207 (shown in red) in chromosome 22.

			*p*-value from Merlin					entry number from LASSO					
				
Markers	Loc (Mb)	LD^a^	E(p)^b^	rank E(p)^c^	E rank(p)^d^	Min.p	Max.p^e^	E(e)	rank E(e)	E rank(e)	Min.e	Max.e	Out^f^
rs131464	23.3948	0	0.075	94	439.6	1.1 × 10^-4^	0.67	1228	205	954.5	56	8702	0
rs133252	24.137	0	0.27	525	1345	7.9 × 10^-4^	0.98	1690	466	1170	32	10001	4
rs5752309	25.0701	0	0.066	74	390.3	6.6 × 10^-6^	0.99	1570	398	915.6	31	10001	11
rs1543335	33.5136	0	0.64	4457	3007.5	5.2 × 10^-2^	1	6452	3757	3113.3	445	10001	82
rs17002034	39.3263	0	0.026	36	184.2	3.3 × 10^-6^	0.48	5127	3167	2499.2	192	10001	71
rs6519313	40.8828	0	0.14	198	743.2	2.8 × 10^-4^	0.95	3431	2009	2021.8	23	10001	20
rs7364152	41.6466	0	0.15	212	785.2	1.2 × 10^-4^	0.85	6747	3873	3293.2	93	10001	73
rs5765113	43.7736	0	1.9 × 10^-3^	20	35.8	7.9 × 10^-8^	0.057	2462	1125	1438.2	200	10001	19
rs6007503	43.989	0	0.069	80	396.5	9.0 × 10^-3^	0.78	2539	1211	1766.3	130	10001	3
rs12159871	47.74	0	0.39	1077	1902	1.4 × 10^-3^	1	8620	4397	3781	1802	10001	141
rs4528878	47.8062	0	0.38	1013	1851.9	0.01	1	5139	3175	2708.2	432	10001	53
rs17013240	48.9997	0	0.56	2735	2671.8	0.016	1	6241	3666	3134.9	529	10001	63
rs5994481	30.8996	0.06	0.016	25	123	5.0 × 10^-6^	0.52	3586	2136	2155.4	15	10001	13
rs136414	30.9051	0.33	4.5 × 10^-6^	6	8.4	1.6 × 10^-13^	1.8 × 10^-4^	3809	2301	2013.1	87	10001	49
rs136416	30.9052	0.32	5.1 × 10^-6^	7	8.8	1.7 × 10^-14^	2.0 × 10^-4^	5726	3452	2751.9	1	10001	77
rs136417	30.9053	0.32	5.2 × 10^-6^	8	9.5	2.3 × 10^-14^	2.0 × 10^-4^	8015	4246	3470.7	3	10001	139
rs136422	30.9064	0.32	6.4 × 10^-6^	9	10.5	5.3 × 10^-14^	2.6 × 10^-4^	6769	3882	3050.7	3	10001	106
rs136457	30.9147	0.22	1.7 × 10^-5^	11	11.9	5.1 × 10^-13^	1.4 × 10^-3^	8810	4429	3750.6	7	10001	159
rs136458	30.9147	0.22	1.7 × 10^-5^	12	12.4	5.1 × 10^-13^	1.4 × 10^-3^	8871	4437	3789	155	10001	158
rs136460	30.9148	0.22	1.6 × 10^-5^	10	11.2	3.7 × 10^-13^	1.4 × 10^-3^	8222	4310	3530.7	8	10001	144
rs136477	30.9184	0.22	2.0 × 10^-5^	14	13.3	2.1 × 10^-12^	1.4 × 10^-3^	4543	2791	2557.2	165	10001	37
rs136485	30.9221	0.22	1.8 × 10^-5^	13	11.7	7.0 × 10^-13^	1.4 × 10^-3^	5008	3092	2649	3	10001	54

^a^LD, linkage disequilibrium between the major causal SNP and other SNPs (measured by *r*^2^)

^b^E(p), averaged *p*-value over 200 replications

^c^rank E(p), rank of the averaged *p*-value over 200 replications

^d^E rank(p), averaged rank of *p*-values (similarly for entry number)

^e^Max.e, 10001 if the SNP was not in any of the first 10000 models

^f^Out, count of replicates for which the SNP was excluded in the LASSO solution path (up to 10,000 models)

### Multi-marker analysis using LASSO

Figure 1B shows LASSO results for Replicate 1 of 200 simulated LDL phenotypes. For Replicate 1, the major causal SNP rs2294207 entered first in the LASSO solution path, which happened in 114 out of 200 replicates. In 84 out of the remaining 86 replicates, one of three nearby SNPs entered first: rs8137034 (42 times), rs2294208 (34 times), and rs5998330 (8 times). Ranks of these four SNPs including the major causal SNP were 5.3, 57.2, 334.1, 1174 on average for 200 replicates (Table 1). Because these nearby SNPs were highly correlated with the causal SNP, once they were included as predictors the causal SNP became a predictor much later (with average rank 5.3). In contrast to single-marker analysis in which the top 15 SNPs with smallest *p*-values were all near the major causal SNP, only 3 SNPs out of these top 15 SNPs were near the major causal SNP and the remaining 12 SNPs were more or less uniformly located (Figure 1B). For Replicate 1 (Figure 1A), 960 SNPs that were excluded from the LASSO analysis (cyan points) included these neighboring SNPs. This was consistent across all 200 replicates: all 14 neighboring SNPs were sometimes excluded from the LASSO solution path. For example, SNP rs136457 was excluded from the LASSO path in 159 out of 200 replicates even though its average rank from single-marker analysis was 11.9 (Table 1). Overall, we have not found much consistency between ranks from Merlin and those from LASSO (correlation = 0.07 across all 200 replicates and correlation = 0.08 in replicate 1, shown in Figure 1C).

## Conclusion

In this paper, we applied single-marker analysis using Merlin and multi-marker analysis using LASSO to the simulated LDL phenotype data on chromosome 22. Single-marker analysis using Merlin correctly provided statistically significant association of the major causal SNP rs2294207 with *p*-value less than 6.9 × 10^-13 ^for all 200 replicates. Multi-marker analysis using LASSO also included this causal SNP as the first predictor in 114 out of 200 replicates, indicating the importance of this SNP. When the causal SNP was not included as the first predictor, one of its three neighboring SNPs was included as the first predictor. Merlin declared statistically significant 14 non-causal neighboring SNPs, whereas the first 10,000 models in the LASSO solution paths often excluded these 14 SNPs. The 12 polygenic SNPs were less statistically significant than these neighboring 14 SNPs by both Merlin and LASSO analyses, indicating that their effects were too small to be detected. Overall, there was little consistency between the rank orders of the 4589 SNPs provided by Merlin and LASSO.

Our results indicate that Merlin and LASSO analyses provide different results. We observe that LASSO typically included 3 SNPs near the causal SNPs out of the 15 SNPs that showed very strong association from Merlin and excluded the remaining SNPs from the LASSO path (up to the first 10,000 models). This may be useful because these neighboring SNPs are not causal. We expected that LASSO would provide better results for the 12 polygenic SNPs. However, this may not have occurred because the strength of their effects was much smaller than the effect of the major causal SNP; thus, for this data set the phenotype appears to be influenced by a single SNP, in which case single-marker analysis will perform better than multi-marker analysis. Hence, our results are inconclusive in terms whether the LASSO analysis provides additional information.

The relative advantage of multi-marker analyses over single-marker will depend on the underlying disease model. Other penalized least-squares methods may provide results more similar to single-marker analysis than LASSO. Ridge regression (penalized regression with L2 penalty) shrinks the coefficients of correlated predictors toward each other, so they borrow strength from each other. In the extreme case of *k *identical predictors, they each get identical coefficients with 1/*k*^th ^the size that any single one would get if fit alone. On the other hand, LASSO (with L1 penalty) is somewhat indifferent to very correlated predictors and will tend to pick one and ignore the rest. The elastic net regression (penalized regression with a convex combination of both penalties) can have the advantages of both ridge and LASSO [8]. We suspect that LASSO may provide better inference for diseases with multiple causal SNPs that are not in LD. For other cases (i.e., diseases with multiple causal SNPs in LD), ridge, elastic net, or haplotype analysis may provide better inference. Further investigation is needed.

## List of abbreviations used

GAW16: Genetic Analysis Workshop 16; LASSO: Least absolute shrinkage and selection operator; LD: Linkage disequilibrium; LDL: Low-density lipoprotein; MAF: Minor allele frequency; SNP: Single-nucleotide polymorphism

## Competing interests

The authors declare that they have no competing interests.

## Authors' contributions

YJS conceived the study, carried out association and LASSO analyses, and drafted the manuscript. TKR carried out all phenotype adjustments. GS developed the concept and set up genotype and map files in the appropriate formats. CCG acquired the data and carried out quality control of the genotypes. DCR participated in the design of the study, helped to draft the manuscript and revised the manuscript. All authors read and approved the final manuscript.
